# High levels of cyclic‐di‐GMP in plant‐associated *P* *seudomonas* correlate with evasion of plant immunity

**DOI:** 10.1111/mpp.12297

**Published:** 2015-10-08

**Authors:** Sebastian Pfeilmeier, Isabel Marie‐Luise Saur, John Paul Rathjen, Cyril Zipfel, Jacob George Malone

**Affiliations:** ^1^ The Sainsbury Laboratory Norwich Research Park Norwich NR4 7UH UK; ^2^ John Innes Centre Norwich Research Park Norwich NR4 7UH UK; ^3^ Research School of Biology The Australian National University Canberra ACT 2601 Australia; ^4^ University of East Anglia Norwich NR4 7TJ UK; ^5^ Present address: Department of Plant–Microbe Interactions Max Planck Institute for Plant Breeding Research D‐50829 Cologne Germany

**Keywords:** cyclic‐di‐GMP, flagellin, immune evasion, *P**seudomonas*, PTI

## Abstract

The plant innate immune system employs plasma membrane‐localized receptors that specifically perceive pathogen/microbe‐associated molecular patterns (PAMPs/MAMPs). This induces a defence response called pattern‐triggered immunity (PTI) to fend off pathogen attack. Commensal bacteria are also exposed to potential immune recognition and must employ strategies to evade and/or suppress PTI to successfully colonize the plant. During plant infection, the flagellum has an ambiguous role, acting as both a virulence factor and also as a potent immunogen as a result of the recognition of its main building block, flagellin, by the plant pattern recognition receptors (PRRs), including FLAGELLIN SENSING2 (FLS2). Therefore, strict control of flagella synthesis is especially important for plant‐associated bacteria. Here, we show that cyclic‐di‐GMP [bis‐(3′‐5′)‐cyclic di‐guanosine monophosphate], a central regulator of bacterial lifestyle, is involved in the evasion of PTI. Elevated cyclic‐di‐GMP levels in the pathogen *P* *seudomonas syringae* pv. *tomato* (*P* *to*) DC3000, the opportunist *P* *. aeruginosa* PAO1 and the commensal *P* *. protegens* Pf‐5 inhibit flagellin synthesis and help the bacteria to evade FLS2‐mediated signalling in *N* *icotiana benthamiana* and *A* *rabidopsis thaliana*. Despite this, high cellular cyclic‐di‐GMP concentrations were shown to drastically reduce the virulence of *P* *to* DC3000 during plant infection. We propose that this is a result of reduced flagellar motility and/or additional pleiotropic effects of cyclic‐di‐GMP signalling on bacterial behaviour.

## Introduction

Plants rely on an innate immune system consisting of passive and active defences to resist potential attack by most microbial pathogens (Dangl *et al*., 2013). During infection, cell surface‐localized pattern recognition receptors (PRRs) perceive pathogen/microbe‐associated molecular patterns (PAMPs/MAMPs), molecules released from microbial pathogens, and trigger local and systemic immune responses (Boller and Felix, 2009). The PRRs FLAGELLIN SENSING2 (FLS2) and EF‐TU RECEPTOR (EFR) specifically recognize the peptidic MAMPs flg22, derived from bacterial flagellin, and elf18, derived from elongation factor thermo‐unstable (EF‐Tu), respectively, and activate an intracellular signal transduction cascade that results in pattern‐triggered immunity (PTI) (Zipfel, 2014). Although flg22 recognition seems to be conserved in most plant species and functional FLS2 orthologues have been identified in many plants, including *Arabidopsis thaliana* (Gomez‐Gomez and Boller, 2000) and *Nicotiana benthamiana* (Hann and Rathjen, 2007), a functional EFR protein has only been found in Brassicaceae (Boller and Felix, 2009; Kunze *et al*., 2004; Zipfel *et al*., 2006). As PTI is activated by features conserved across entire groups of microbes, it is sufficient to ward off a broad range of microbial threats (Boller and Felix, 2009; Zipfel, 2014).

The molecular events that occur on MAMP recognition have been studied extensively, especially using the flg22/FLS2 and elf18/EFR perception systems. On ligand perception, both FLS2 and EFR form heteromeric complexes with the receptor‐like kinase BAK1/SERK3 and the related protein BKK1/SERK4 (Chinchilla *et al*., 2007; Heese *et al*., 2007; Roux *et al*., 2011; Schulze *et al*., 2010). BAK1 is a key co‐receptor, mediating full signal activation of downstream responses through auto‐ and trans‐phosphorylation events (Cao *et al*., 2013; Schulze *et al*., 2010; Schwessinger *et al*., 2011; Sun *et al*., 2013; Wang *et al*., 2014; Yan *et al*., 2012). Activation of FLS2 and EFR converges on signalling pathways that share numerous downstream elements and ultimately induce an array of defence responses, including reactive oxygen species (ROS) production, callose deposition, stomatal closure and transcriptional reprogramming (Boller and Felix, 2009; Macho and Zipfel, 2014). The MAMP‐triggered oxidative burst, a hallmark of PTI (Boller and Felix, 2009), is characterized by a rapid and transient apoplastic ROS production by the NADPH oxidases RBOHD in *A. thaliana* and RBOHB in *N. benthamiana* (Marino *et al*., 2012; Nuhse *et al*., 2007; Segonzac *et al*., 2011; Zhang *et al*., 2007).

Plants are continuously exposed to a highly complex microbial community that contains not only pathogens, but also many commensal and beneficial species. Indeed, the microbiomes of the phyllosphere and rhizosphere play critical roles in the adaptation of a plant to its environment (Berendsen *et al*., 2012; Guttman *et al*., 2014). Flagellin and EF‐Tu (with the immunogenic epitopes flg22 and elf18, respectively) are typical MAMPs. They are widespread among many bacterial species, abundant and highly conserved, fulfilling important bacterial functions (Pel and Pieterse, 2013). Although the PRRs for flg22 and elf18 ensure the reliable recognition of diverse bacterial pathogens, the universal nature of these MAMPs means that beneficial and commensal microbes are also potentially exposed to immune recognition (van Loon, 2007; Van Wees *et al*., 2008; Zamioudis and Pieterse, 2012). Indeed, some symbiotic bacteria, such as *Rhizobium*, have evolved to evade flg22 recognition (Felix *et al*., 1999).

*Pseudomonas* is an important genus of plant‐associated bacteria that contains both phytopathogenic and commensal species. *Pseudomonas syringae* pv. *tomato* (*Pto*) DC3000 is a foliar, hemibiotrophic plant pathogen that causes bacterial speck disease on tomato and *A. thaliana* (Xin and He, 2013). Primary infection sites are natural openings and wounds, through which bacteria migrate into the apoplast before multiplying rapidly, leading to chlorosis and necrosis of plant tissue (Xin and He, 2013). *Pseudomonas aeruginosa* is an opportunistic pathogen that infects immune compromised humans and can also colonize and infect plants, although this ability is limited to specific hosts (Starkey and Rahme, 2009). Attachment, colonization and proliferation of *P. aeruginosa* in *A. thaliana* have been described as having similarities to the *Pto* DC3000 infection process (Plotnikova *et al*., 2000), although no specific plant virulence factor has been identified. *Pseudomonas aeruginosa* growth in the intercellular space leads to systemic infection and, ultimately, to severe soft‐rot symptoms (Plotnikova *et al*., 2000). Conversely, *Pseudomonas protegens* Pf‐5 is a soil bacterium that colonizes the rhizosphere and promotes plant growth by suppression of a wide variety of plant diseases over a broad host range (Loper *et al*., 2007). *Pseudomonas protegens* Pf‐5 produces multiple secondary metabolites, including pyoluteorin and 2,4‐diacetylphloroglucinol, that underpin its biocontrol capacities. In addition to the production of antibiotics, siderophore secretion and genetic features, such as broad catabolic pathways, an expanded array of efflux systems and numerous genes conferring tolerance to oxidative stress enable *P. protegens* Pf‐5 to cope with environmental stress and microbial competition in the rhizosphere. Consistent with its commensal lifestyle, certain pathogenicity factors, such as the type‐III secretion system (T3SS), are not present in the Pf‐5 genome (Loper *et al*., 2007, 2012). Like many Gram‐negative bacteria, these three plant‐associated *Pseudomonas* spp. have polar flagella that confer directed mobility and enable both the spatial colonization of plant surfaces and migration into the apoplast (Jackson, 2009).

The bacterial second messenger cyclic‐di‐GMP (cdG) is a key regulator of flagella expression and function in *Pseudomonas* (Hickman and Harwood, 2008). In general, cdG controls processes involved in the switch between single‐celled motile and communal sessile lifestyles in many bacterial species (Hengge, 2009). *Pseudomonas* and other bacteria integrate environmental cues and intracellular signals in cdG signalling pathways, which regulate a diverse range of behaviours, including motility (Dasgupta *et al*., 2003), adhesion to surfaces (Newell *et al*., 2011), biofilm formation (Hickman *et al*., 2005) and virulence (Kulasakara *et al*., 2006). The level of intracellular cdG is coordinated by the opposing enzymatic activities of multiple diguanylate cyclases (DGCs) and phosphodiesterases (PDEs) (Hengge, 2009). In *P. aeruginosa*, flagella‐driven motility and exopolysaccharide (EPS) production are reciprocally controlled by several cdG‐dependent systems (Dasgupta *et al*., 2003; Lee *et al*., 2007), with the cdG‐binding transcription factor FleQ playing a central role. FleQ is a σ^54^‐dependent master regulator that controls the expression of genes, including the *fleSR* two‐component system, flagella export apparatus loci and genes involved in the initiation of flagella basal body assembly (Dasgupta *et al*., 2003; Robleto *et al*., 2003). When cdG levels are low, FleQ inhibits EPS production by the repression of *pel* and *psl* EPS‐operon transcription, but is required for the transcription of multiple flagellar genes. Correspondingly, on binding of cdG, FleQ both releases EPS biosynthetic gene repression and abolishes flagella gene transcription, enabling the switch from motility to EPS production and biofilm formation (Baraquet and Harwood, 2013; Hickman and Harwood, 2008). Homologues of FleQ are present in most *Pseudomonas* spp., including *P. syringae* and *P. protegens* (Winsor *et al*., 2011).

Here, we investigate the impact of increased intracellular cdG levels in different *Pseudomonas* spp. on plant immune responses. Bacterial extracts from the opportunistic *P. aeruginosa* PAO1, the commensal *P. protegens* Pf‐5 and the pathogenic *Pto* DC3000 were assessed for their ability to elicit plant defence responses by measuring ROS production in *A. thaliana* and *N. benthamiana* leaves. Overexpression of the constitutively active DGC *wspR19* (Goymer *et al*., 2006) increased cdG levels in the extracts of all three *Pseudomonas* spp., leading to a strongly suppressed ROS response in each case. This effect was almost entirely dependent on reduced signalling through the FLS2 receptor, a finding consistent with greatly reduced flagellin (FliC) levels seen on cdG overproduction in each case. However, although increased cdG levels led to reduced *fliC* expression and contributed to the evasion of FLS2‐mediated immunity, *A. thaliana* infection by *Pto* DC3000 was severely compromised on DGC overexpression. We show that high cdG levels in plant‐associated *Pseudomonas* spp. suppress flagellin expression, which correlates with evasion of the FLS2‐mediated immune response. However, any advantage this might confer during plant infection is overwhelmed by the pleiotropic effects of cdG on other virulence‐associated phenotypes.

## Results

### Increased *P* *seudomonas* cdG levels lead to a suppressed ROS response in *N* *. benthamiana* and *A* *. thaliana*

In order to examine the effect of increased cdG levels on the induction of plant immune responses by *Pseudomonas* spp., we first produced a broad‐host‐range expression vector for a constitutively active allele of the DGC *wspR* (pBBR2/5‐*wspR19*), and transformed the three species included in this study (*Pto* DC3000, *P. protegens* Pf‐5 and *P. aeruginosa* PAO1). In agreement with previous findings (Goymer *et al*., 2006), *wspR19* expression resulted in aggregative, wrinkled colony morphologies, enhanced Congo Red dye binding, which indicates the production of polysaccharides, such as cellulose, and reduced motility (Fig. S1, see Supporting Information). Overexpression of DGC genes *in trans* leads to the intracellular accumulation of cdG in various bacterial species (Hengge, 2009). Accordingly, the expression of *wspR19* led to increased cdG levels in all three *Pseudomonas* spp., as determined by liquid chromatography‐tandem mass spectrometry (LC‐MS/MS) analysis (Table 1). The cdG levels measured for the wild‐type (WT) containing the empty vector and for the DGC‐overexpressing strains (*wspR19*) of *P. protegens* Pf‐5 and *P. aeruginosa* PAO1 were comparable with the levels described previously for PAO1 (Malone *et al*., 2010). Interestingly, *wspR19* expression increased cdG concentration to a much lesser extent in *Pto* DC3000 compared with the increases seen in *P. protegens* Pf‐5 and *P. aeruginosa* PAO1.

**Table 1:** Expression of *wsp* *R* *19* in *Pseudomonas* elevates cellular cyclic‐di‐GMP concentrations

Species		[cyclic‐di‐GMP] (pmol/mg of bacterial protein)	
*Pseudomonas syringae* pv. *tomato* DC3000	WT	1.97	± 1.66
	*wspR19*	32.85**	± 3.12
*Pseudomonas protegens* Pf‐5	WT	1.70	± 0.06
	*wspR19*	206.02***	± 5.67
*Pseudomonas aeruginosa* PAO1	WT	2.45	± 0.67
	*wspR19*	223.49***	± 13.32

Data represent the means of two biological replicates with ‘±’ representing the standard error (*n* = 2). Asterisks indicate statistical significance (**P < 0.01; ***P < 0.001) between wild‐type (WT) and *wspR19* extracts based on an unpaired Mann–Whitney test.

**Figure 1:**
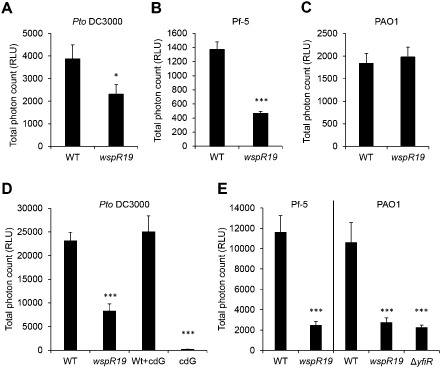
Extracts from *P* *seudomonas* bacteria with high cyclic‐di‐GMP levels trigger reduced reactive oxygen species (ROS) burst. Total ROS accumulation in *A* *rabidopsis thaliana* (A–C) and *N* *icotiana benthamiana* (D, E) leaf discs after treatment with *Pseudomonas* extracts, expressed as relative light units (RLU) over 60 min. Leaf discs were treated with extracts from *Pseudomonas syringae* pv. *tomato* (*Pto*) DC3000 (A, D), *P* *. protegens* Pf‐5 (B, E) or *P* *. aeruginosa* PAO1 (C, E) carrying empty *p* *BBR* *2* vector (WT), *p* *BBR* *‐wsp* *R* *19* (*wspR19*), PAO1 Δ*yfi* *R* or 1 μm purified cdG. Values are means ± standard error (*n* = 8). Asterisks indicate statistically significant difference (*P < 0.05; ***P < 0.001) between treatment of wild‐type extracts and other samples based on a two‐tailed Mann–Whitney test.

Next, we investigated the effect of high cdG levels in *Pseudomonas* on the induction of PTI in different plants by measuring the ROS burst on the application of bacterial extracts. The extracts of *Pto* DC3000 and *P. protegens* Pf‐5 overexpressing the DGC *wspR19* triggered a reduced ROS burst in both *A. thaliana* and *N. benthamiana* compared with WT extracts (Fig. 1A,B,D,E), whereas extracts of *P. aeruginosa* PAO1 expressing *wspR19* only caused a reduced ROS in *N. benthamiana* (Fig. 1E), but not *A. thaliana* (Fig. 1C). To test whether the cdG molecule itself is responsible for the suppression of ROS responses, we added chemically synthesized cdG to the *Pto* DC3000 bacterial extract. No difference in the *A. thaliana* ROS response was seen compared with the *Pto* DC3000 WT extract alone. Likewise, we saw no ROS response on addition of 1 μm cdG alone (Fig. 1D). Bacterial extract from a previously characterized *P. aeruginosa* PAO1 mutant with increased intracellular cdG levels, Δ*yfiR* (Malone *et al*., 2010), triggered a similarly reduced ROS response to the PAO1 *wspR19‐*overexpressing strain (Fig. 1E). Thus, we conclude that the intracellular activity of cdG in the bacterium leads to loss of ROS production triggered by the tested bacterial extracts in *N. benthamiana*, and in *A. thaliana* for *Pto* DC3000 and Pf‐5 extracts.

### FLS2‐mediated ROS is specifically reduced by *wsp* *R* *19* expression

The ROS burst can be initiated by the activation of several different PRRs (Macho and Zipfel, 2014). As the bacterial extracts used in our experiments are made from whole‐cell lysates, they may contain numerous different MAMPs. Consequently, we tested whether the suppressed ROS bursts seen for the *wspR19* extracts depend on one or more specific plant receptors.

**Figure 2:**
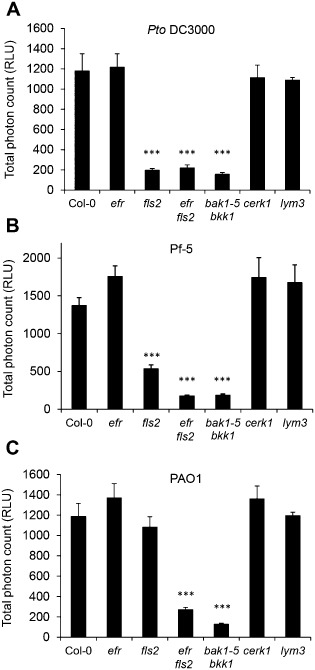
Flagellin and elongation factor thermo‐unstable (EF‐Tu) in *P* *seudomonas* extracts are the main elicitors of reactive oxygen species (ROS) production in *A* *rabidopsis*. Total ROS accumulation was measured in *A* *rabidopsis* genotypes Col‐0, *efr*, *fls2*, *efr fls2*, *bak1‐5 bkk1*, *cerk1‐2* and *lym3‐1* (A–C). The ROS burst was induced by extracts from *Pseudomonas syringae* pv. *tomato* (*Pto*) DC3000 (A), *P* *. protegens* Pf‐5 (B) or *P* *. aeruginosa* PAO1 (C). Values are means ± standard error (*n* = 8). Asterisks indicate statistically significant difference (***P < 0.001) between the treatment of wild‐type extracts and other samples based on a two‐tailed Mann–Whitney test. RLU, relative light units.

To identify the major eliciting agents in our bacterial extracts, we characterized the ROS response produced by *Pseudomonas* WT extracts on a series of different *A. thaliana* PRR knock‐out mutants. The ROS response of *efr*, *cerk1‐2* and *lym3‐1* plants (CERK1 and LYM3 are involved in bacterial peptidoglycan perception; Willmann *et al*., 2011) to all tested extracts was comparable with that of Col‐0 (Fig. 2). Conversely, total ROS accumulation was drastically reduced in *fls2* and even more so in *efr fls2* and *bak1‐5 bkk1* plant lines (relative to Col‐0) after application of WT extracts from *Pto* DC3000 and *P. protegens* Pf‐5 (Fig. 2A,B). This suggests that the ROS signal is mainly triggered by flagellin and, to a lesser extent, by EF‐Tu for these two extracts. WT *P. aeruginosa* PAO1 extract triggered equally high ROS production in Col‐0, *efr* and *fls2* plants, whereas the ROS response was strongly diminished in *efr fls2* and *bak1‐5 bkk1* (Fig. 2C). Previous work has shown that flg22‐ or elf18‐triggered ROS production is drastically reduced in *bak1‐5 bkk1* because of the role of BAK1 and BKK1 as co‐receptors for FLS2 and EFR (Chinchilla *et al*., 2007; Heese *et al*., 2007; Roux *et al*., 2011; Sun *et al*., 2013). Furthermore, the signal transduction pathways of both receptors converge into the same signalling pathway, which means that the intensity of FLS2 and EFR activation is not additive and both elicitors can compensate for each other (Macho and Zipfel, 2014). Our results suggest that, under the culture conditions used here, the main elicitor in *Pto* DC3000 and *P. protegens* Pf‐5 extracts is flagellin, whereas, in the *P. aeruginosa* PAO1 extract, flagellin and EF‐Tu are equally strong elicitors.

**Figure 3:**
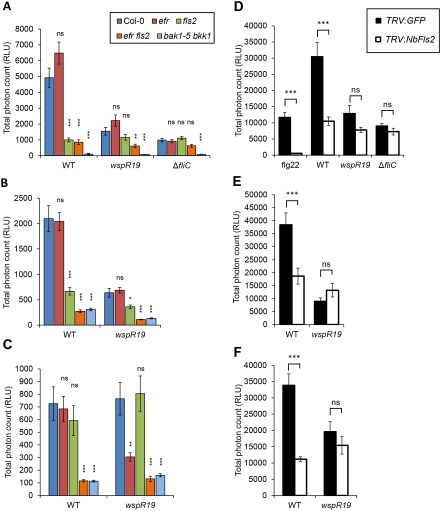
Reduced reactive oxygen species (ROS) burst triggered by *Pseudomonas* extracts with high cyclic‐di‐GMP is FLAGELLIN SENSING2 (FLS2) dependent. Total ROS accumulation was measured in *A* *rabidopsis thaliana* genotypes Col‐0, *efr*, *fls2*, *efr fls2*, *bak1‐5 bkk1* (A–C) and *NbFls2*‐silenced (*TRV*:*NbF* *ls2*) or control silenced (*TRV*:*GFP*) *N* *icotiana benthamiana* plants (D–F). The ROS burst was induced by extracts from *Pseudomonas syringae* pv. *tomato* (*Pto*) DC3000 (A, D), *P* *. protegens* Pf‐5 (B, E) or *P* *. aeruginosa* PAO1 (C, F) expressing *wsp* *R* *19* and their respective control strains carrying empty *p* *BBR* *2* vector (WT) or *P* *to* DC3000 Δ*fli* *C*. Values are means ± standard error (*n* = 8). Asterisks indicate statistically significant difference (*P < 0.05; **P < 0.01; ***P < 0.001) between *A* *. thaliana* Col‐0 and other transgenic or mutant lines, or between *N* *. benthamiana* control plants and *NbF* *ls2*‐silenced plants, based on a two‐tailed Mann–Whitney test. RLU, relative light units.

Next, we repeated the ROS burst experiments for the *A. thaliana* PRR knock‐out mutants with extracts from *wspR19*‐overexpressing strains. Overexpression of *wspR19* in *Pto* DC3000 and *P. protegens* Pf‐5 suppresses the ROS burst elicited by their extracts in *A. thaliana* Col‐0 and the *efr* knock‐out mutant (Fig. 3A,B). Because the major elicitor of ROS production in the extracts of these two species is probably flagellin (Fig. 2A,B), this suggests that suppression of the ROS response by *wspR19* expression is a result of decreased flagellin perception by FLS2. Extracts from a *Pto* DC3000 Δ*fliC* strain were tested as a control. *Pto* DC3000 *wspR19* extracts induced slightly higher ROS production in Col‐0 and *efr* plants compared with extracts from the Δ*fliC* strain, but ROS was equally low in *fls2* and *efr fls2* (Fig. 3A). The slightly higher ROS production for *wspR19* extracts in plants containing the functional FLS2 receptor is probably the consequence of residual flagellin levels in these strains. *Pseudomonas aeruginosa* PAO1 *wspR19*‐expressing extracts induced equally strong ROS production in Col‐0 and *fls2*, whereas the ROS response was reduced in *efr* plants, in which ROS production mainly derives from activation of the FLS2 pathway (Fig. 3C). The perception of flagellin and EF‐Tu by FLS2 and EFR, respectively, has been extensively studied in *A. thaliana* (Boller and Felix, 2009); there is no current evidence for other *A. thaliana* receptors recognizing flagellin or EF‐Tu, nor for the existence of additional FLS2 or EFR ligands. As the presence of either flagellin or EF‐Tu in the PAO1 extracts seems to be sufficient to trigger the full ROS response in the plant (Fig. 2C), ROS production is only reduced if elements of both recognition systems are simultaneously absent. Thus, the reduction in ROS production on *wspR19* expression in *P. aeruginosa* PAO1 also appears to be FLS2 dependent.

To examine whether the effect of *wspR19* expression on ROS accumulation also depends on FLS2 in *N. benthamiana*, we silenced *NbFls2* using virus‐induced gene silencing. *NbFls2* silencing was confirmed by abolished ROS production on application of 10 nm flg22 peptide (Fig. 3D). ROS accumulation on treatment with WT extracts from all three *Pseudomonas* spp. was strongly reduced in *NbFls2*‐silenced plants (*TRV*:*NbFls2*) compared with mock‐silenced control plants (*TRV*:*GFP*) (Fig. 3D–F), indicating that, within the bacterial extracts, flagellin is the major elicitor of the ROS burst in *N. benthamiana*. *Nicotiana benthamiana* is unable to perceive EF‐Tu, as it lacks a functional EFR homologue (Zipfel *et al*., 2006). The suppressive effect of *wspR19* expression is dependent on FLS2, as there was no significant difference in the ROS signal between *TRV*:*NbFls2* and *TRV*:*GFP* plants when the *wspR19*‐expressing extracts were tested (Fig. 3D–F). Together, these results indicate that the reduced *A. thaliana* and *N. benthamiana* ROS responses induced by extracts of *Pseudomonas* spp. with increased cdG levels are dependent on signalling through the FLS2 receptor.

### Expression of *wsp* *R* *19* leads to impaired accumulation of flagellin

**Figure 4:**
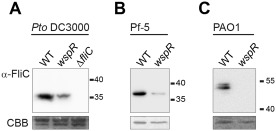
High cyclic‐di‐GMP levels suppress FliC accumulation in *P* *seudomonas*. Extracts from *Pseudomonas syringae* pv. *tomato* (*P* *to*) DC3000 (A), *P* *. protegens* Pf‐5 (B) and *P* *. aeruginosa* PAO1 (C) carrying the empty *p* *BBR* *2* vector (WT) or *p* *BBR* *‐wsp* *R* *19* (*wsp* *R* *19*) and *P* *to* DC3000 Δ*fli* *C* were analysed by Western blot using α‐flagellin antibody. Membranes were stained with Coomassie Brilliant Blue (CBB) as loading controls.

Our data from the ROS assays in *A. thaliana* and *N. benthamiana* indicated that *wspR19* expression in the three tested *Pseudomonas* spp. affected the amount of the MAMP flagellin in the extracts, thus reducing the FLS2‐induced ROS burst relative to WT. We tested the accumulation of flagellin in the bacterial extracts by Western blot analysis using a commercial anti‐FliC antibody. Flagellin accumulation was drastically reduced in *Pto* DC3000 and *P. protegens* Pf‐5 (Fig. 4A,B) and undetectable in *P. aeruginosa* PAO1 (Fig. 4C) on *wspR19* expression compared with the respective WT extracts. Studies in *P. aeruginosa* have shown that cdG negatively affects the expression of flagella genes by binding to FleQ, a master regulator of flagella gene transcription (Baraquet and Harwood, 2013; Dasgupta *et al*., 2003; Hickman and Harwood, 2008). This would explain the reduced accumulation of flagellin in the *wspR19‐*expressing extracts compared with WT strains, and the correspondingly weaker ROS response as a result of reduced FLS2 activation (Fig. S2, see Supporting Information).

To examine whether the down‐regulation of flagella synthesis through the elevation of cdG levels is a strategy adopted by *Pto* DC3000 on plant colonization, we attempted to extract and measure cdG from *Pto* DC3000 growing *in planta*. Unfortunately, despite our best efforts and the examination of large amounts of infected plant tissue, we were unable to reliably quantify *in planta* cdG levels. Although this remains a highly interesting question, it appears that *in planta* cdG measurements are currently beyond the limit of our technical capabilities.

### Virulence of *P* *to* DC3000 during infection is drastically reduced by *wsp* *R* *19* expression

**Figure 5:**
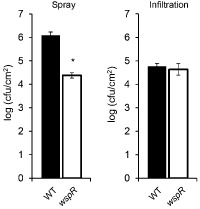
*wsp* *R* *19* expression reduces the virulence of *Pseudomonas syringae* pv. *tomato* (*P* *to*) DC3000 during plant infection after spray inoculation, but not after infiltration. Growth of *P* *to* DC3000 carrying empty *p* *BBR* *2* vector (WT) or *p* *BBR* *‐wsp* *R* *19* (*wsp* *R* *19*) at 2 days post‐infection of *A* *rabidopsis thaliana* Col‐0 plants. Bacteria were either sprayed onto [inoculum 2.5 × 10^7^ colony‐forming units (cfu)/mL] or syringe infiltrated into (inoculum 10^5^ cfu/mL) the plant. Values are means ± standard error (*n* = 4). Significant differences (*P < 0.05) based on two‐tailed Mann–Whitney test.

As FLS2‐mediated flg22 perception impedes the success of bacterial infection (Forsyth *et al*., 2010; Hann and Rathjen, 2007; Zeng and He, 2010; Zipfel *et al*., 2004), we tested the effect of *wspR19* expression on the virulence of *Pto* DC3000 during the infection of *A. thaliana* plants. The growth of *Pto* DC3000 *wspR19* was drastically reduced after spray infection of *A. thaliana* Col‐0 (Fig. 5). This suggests that high intracellular cdG levels exert a strong negative effect on *Pto* DC3000 virulence, which, in turn, cancels out any benefit from the evasion of FLS2‐triggered immunity. FLS2‐induced defences against the virulent *Pto* DC3000 may only be effective when the flagellin‐triggered immune responses are activated in the early stages of the colonization process (Zipfel *et al*., 2004). In order to dissect the different effects of *wspR19* overexpression on plant infection, we syringe infiltrated *Pto* DC3000 WT and the *wspR19*‐expressing strains directly into the apoplast, bypassing the critical initial colonization steps. Interestingly, the *Pto* DC3000 and *wspR19*‐overexpressing strains were equally virulent after infection by infiltration (Fig. 5), suggesting that the infectious disadvantage arising from increased cdG levels is associated exclusively with the initial stages of plant colonization and migration into the apoplast. We also tested the effect of high cdG levels on the virulence of *P. aeruginosa* PAO1 during infection of *A. thaliana*. However, *P. aeruginosa* PAO1 did not proliferate or induce any disease symptoms in the *A. thaliana* ecotypes Col‐0, Wassilewskija (Ws‐0) or Llagostera (Ll‐0) (data not shown). Previously, plant infections with *P. aeruginosa* have been described for strain PA14, which contains additional pathogenicity islands that might determine its virulence in *A. thaliana* (Plotnikova *et al*., 2000).

## Discussion

Plants sense and respond to the presence of pathogenic microbes by the recognition of MAMPs, ubiquitous microbe‐associated molecules, using plasma membrane‐localized PRRs. MAMP perception triggers an intracellular signal transduction cascade and a subsequent defence response (Boller and Felix, 2009). Flagellin, the central structural unit of the flagellum filament, is a strong immunogen that elicits PTI in plants (Boller and Felix, 2009). Our experiments, measuring ROS production in *A. thaliana* and *N. benthamiana* leaves after application of whole‐cell extracts, revealed that flagellin from *Pto* DC3000, *P. protegens* Pf‐5 and *P. aeruginosa* PAO1 contributes substantially to the induction of this early plant immune response.

The flagellum is an important bacterial organelle, enabling the bacterium to sense and explore its environment, and contributing to the initial attachment to surfaces (Rossez *et al*., 2015). Flagella‐driven motility plays a critical role in both effective plant infection and commensal rhizosphere colonization. It facilitates colonization of plant surfaces, migration into the apoplast and movement through the soil towards the nutrient‐rich environment of the plant root (Lugtenberg *et al*., 2001). The role of flagella during plant interactions is therefore somewhat ambiguous. Plant‐associated bacteria must balance the requirement for flagella‐driven motility in colonization and infection with the downside of potential immune recognition.

To successfully colonize and infect plants, both pathogenic and commensal bacteria need to evade recognition, or to suppress host immune responses. Bacteria have evolved a variety of mechanisms to evade flg22‐triggered immunity (Rossez *et al*., 2015). One important strategy for evading plant immune recognition is the control of flagella synthesis. Bacteria can down‐regulate or switch off flagella expression during infection when motility is unnecessary. Although the expression of flagella genes is controlled by a number of different signal inputs, the transcriptional regulator FleQ plays a central role in this process via cdG (Baraquet and Harwood, 2013; Hickman and Harwood, 2008), with FleQ inactivation by cdG binding leading to the down‐regulation of flagella gene expression in *Pseudomonas* spp. (Baraquet and Harwood, 2013). Here, we suggest a direct link between cdG‐regulated flagella synthesis and evasion of plant immunity. This relationship is widespread, and applies to commensal as well as specific and non‐specific plant pathogens. Elevated cdG levels in *Pto* DC3000, *P. protegens* Pf‐5 and *P. aeruginosa* PAO1 reduce extracellular flagellin levels, and thus help the bacteria to evade the FLS2‐mediated immune response in *N. benthamiana* and *A*. *thaliana* plants. Reduced flagellin levels appear to explain most, if not all, of the ROS‐suppressive effect of cdG overproduction, as little or no additional effect was observed on *wspR19* expression in either *fls2* or *efr fls2* plants. Although flagellin is a major elicitor in the bacterial extracts from all three *Pseudomonas* spp., EF‐Tu appears to be a stronger elicitor in *P. aeruginosa* PAO1 extracts relative to *Pto* DC3000 and *P. protegens* Pf‐5. MAMPs from different species generally vary in their eliciting potential (Clarke *et al*., 2013; Lacombe *et al*., 2010) as a result of allelic variation in the recognized epitope. The immunogenic elf18 sequences from *P. protegens* Pf‐5 and *P. aeruginosa* PAO1 are identical, which suggests that the difference in their ability to trigger a ROS burst might be a result of post‐translational modifications or protein abundance in the extract.

cdG regulates various phenotypic output pathways that define the bacterial lifestyle, and is consequently likely to play an important role in controlling the association of bacteria with plants. This relationship is indirect, and relates entirely to the control of bacterial phenotypes. In contrast with the mammalian innate immune system in which a specific immune receptor for cdG has been found (Burdette *et al*., 2011), no direct effect of cdG on plant immunity has been reported to our knowledge, and purified cdG did not affect plant ROS production in our experiments. Recently, researchers have started to examine the role of cdG signalling in the interactions of commensal and pathogenic *Pseudomonas* spp. with their host plants. The DGCs WspR and Rup4959 play important roles in effective wheat rhizosphere colonization by the commensal species *P. fluorescens* F113 and *P. putida* KT2440, respectively (Barahona *et al*., 2011; Matilla *et al*., 2011). In *P. syringae*, artificial elevation of cdG levels by overexpression of a DGC gene induces pleiotropic responses, including reduced motility, increased EPS production and enhanced biofilm formation, which together produce aberrant plant interaction phenotypes (Perez‐Mendoza *et al*., 2014). More specifically, deletion of the putative PDE gene *bifA* results in both decreased motility and reduced virulence in *Pto* DC3000 (Aragon *et al*., 2015). Highlighting the relevance of cdG for bacterial virulence, the DGC Chp8 is part of the *hrp* regulon, a gene cluster containing the T3SS and expressed in response to plant signals in *Pto* DC3000 (Engl *et al*., 2014). Overexpression of Chp8 produces high cellular cdG levels, once again leading to EPS production and reduced flagella expression (Engl *et al*., 2014).

Overexpression of a DGC *in trans* drastically changed the global cdG concentration in all three bacterial species tested here. In turn, increased cdG levels trigger significant changes in the various signalling networks controlled by this important second messenger (Hengge, 2009). This is borne out by the major shifts in colony morphology, polysaccharide production and motility seen for the *wspR19‐*overexpressing strains. Despite these pleiotropic effects, our research shows that the suppressive effect of cdG on the plant immune response depends almost entirely on the reduced signal transduction through the FLS2 receptor, as a consequence of reduced flagellin production in the cdG‐overproducing strains.

Although cdG overproduction enables bacteria to evade FLS2‐mediated immunity, high levels of cdG actually lead to drastically reduced *Pto* DC3000 virulence during *A. thaliana* infection. We propose that the reduction in virulence on *wspR19* expression is largely a result of the loss of flagella‐driven motility. Restriction of bacterial growth as a result of flagellin recognition in *A. thaliana* is highly effective against non‐adapted pathogens (Forsyth *et al*., 2010; Li *et al*., 2005; de Torres *et al*., 2006; Zeng and He, 2010). Conversely, against adapted pathogens, such as *Pto* DC3000, FLS2‐mediated immunity is only effective when the bacteria are detected in the early stages of infection (Hann and Rathjen, 2007; Zeng and He, 2010; Zipfel *et al*., 2004). During these initial infection steps, bacteria must trade off the evasion of FLS2‐mediated immune responses with the loss of flagellar motility. It has been reported that the virulence of a *Pto* DC3000 Δ*fliC* mutant is compromised on spray infection in *A. thaliana* Col‐0, but that the mutant grows as well as WT when the bacteria are syringe infiltrated into the apoplast (Clarke *et al*., 2013; Li *et al*., 2005). Likewise, in our experiments, overexpression of *wspR19* negatively affected bacterial growth only during spray infections, in which bacteria have to attach to the plant surface and migrate into the apoplast, but had no effect on virulence following leaf infiltration.

In addition to the loss of flagellar motility, other pleiotropic effects of cdG signalling may contribute to the compromised virulence of *Pto* DC3000 overexpressing *wspR19*. Thus, infiltration of bacteria directly into the apoplast might also bypass these virulence‐associated cdG pathways. These include a reduction in pili‐driven motility (Kazmierczak *et al*., 2006), interference with the correct deployment and function of the T3SS (Kulasakara *et al*., 2006), overproduction of EPS or other attachment factors in an inappropriate context (Gal *et al*., 2003) and as yet undefined effects on secondary metabolism and small molecule secretion (Malone *et al*., 2010). Clearly, the negative effects of high cdG levels are far less severe during an established infection, although whether the lifestyle transition from colonization and initial infection to apoplastic proliferation is accompanied by a significant increase in the intracellular level of cdG in *Pto* DC3000 remains to be determined. Here, we established a potential role for the bacterial second messenger cdG in plant immune evasion, and showed that this effect is mediated by a reduction in the levels of the MAMP flagellin, and consequently a reduced FLS2‐mediated host immune response. However, bacteria cannot simply overproduce cdG whenever they encounter host plants; the complex intracellular signalling networks controlled by cdG play important roles in mediating the initial stages of plant infection, and flagella‐driven motility appears to be at least as important to infection as immune system evasion, at least until the infection is established.

## Experimental Procedures

### Plants and growth conditions

All plants were grown in soil in single pots in controlled environment rooms at 22 °C with a 10‐h (*A. thaliana*) or 16‐h (*N. benthamiana*) light period. The mutants and transgenic lines used in this study were produced in the background of the *A. thaliana* ecotype Columbia (Col‐0) and have been described previously as follows: *efr‐1* (Zipfel *et al*., 2006), *fls2c* (Zipfel *et al*., 2004), *efr fls2* (Zipfel *et al*., 2006), *bak1‐5 bkk1‐1* (Roux *et al*., 2011; Schwessinger *et al*., 2011), *lym3‐1* (Willmann *et al*., 2011) and *cerk1‐2* (Miya *et al*., 2007).

### Microbial strains and growth conditions

*Pseudomonas syringae* pv. *tomato* (*Pto*) DC3000 was grown in King's medium B (KB) at 28 °C, Pf‐5 in lysogenic broth (LB) at 28 °C and PAO1 in LB at 37 °C. *Pseudomonas* spp. were transformed by electroporation. The *wspR19* allele was obtained from *P. fluorescens* SBW25 *wspR* carrying a R129C mutation resulting in constitutive DGC activity (Goymer *et al*., 2006). *wspR19* was cloned into a broad‐host‐range pBBR‐MCS2/5 (pBBR2/5) plasmid (Kovach *et al*., 1995) between the *Bam*HI and *Eco*RI sites. *Pto* DC3000 and Pf‐5 were transformed with pBBR2*‐wspR19* or pBBR2 empty vector and grown in medium supplemented with 25 μg/mL kanamycin. *Pseudomonas aeruginosa* PAO1 containing pBBR5‐*wspR19* or the pBBR5 empty vector was grown in medium supplemented with 30 μg/mL gentamycin.

### Phenotypic tests

Colony morphologies were examined for 5‐μL spots of *Pseudomonas* overnight cultures, after overnight incubation at 28 °C on 1.3% agar minimal medium (M9 salts, 0.4% pyruvate) plates containing 0.004% Congo Red dye. Photographs were taken with a Leica M165 FC microscopy system (Leica, Wetzlar, Germany). To measure swimming motility, 0.3% KB agar plates containing the appropriate antibiotics were poured and allowed to set and dry for 1 h in a sterile flow chamber. Plates were inoculated with 2‐μL spots of *Pseudomonas* overnight cultures, and incubated for 2 days at room temperature.

### Preparation of bacterial extracts

Bacteria were grown on KB or LB plates (1.3% agar) containing the appropriate antibiotics. *Pto* DC3000 and *P. protegens* Pf‐5 were grown at 28 °C for 21 h and 18 h, respectively, and *P. aeruginosa* PAO1 at 37 °C for 10 h. Bacteria were scraped off the plates and resuspended in sterile demineralized water. Cells were lysed by boiling at 95 °C for 15 min with intermediate vortexing and immediately placed on ice. Bacterial extract was obtained by centrifugation of the lysed cells for 10 min at 16 000 g at 4 °C, and the supernatant was collected for further analysis. Bacterial extracts were normalized to 5 μg/mL total protein concentration determined using the Bradford assay (BioRad, Hercules, CA, USA).

### Bacterial infections

*Arabidopsis thaliana* Col‐0 and *fls2* plants were grown for 4–5 weeks. *Pto* DC3000 cultures were grown overnight at 28 °C in liquid KB medium containing 25 μg/mL kanamycin to an optical density at 600 nm (OD_600_) between 0.6 and 1.0. Bacteria were resuspended in 10 mm MgCl_2_ and adjusted to OD_600_ = 0.0002 [10^5^ colony forming units (cfu)/mL] for syringe infiltration and to OD_600_ = 0.05 (2.5 × 10^7^ cfu/mL) for spray infection. Shortly before spraying, 0.02% Silwet L‐77 (Lehle Seeds, Round Rock, TX, USA) was added to the suspension. Plants were sprayed until run‐off and covered during the first day of infection. Four different plants per bacterial strain were infected and two leaf discs (7 mm in diameter) per plant were collected at 2 days post‐infection in 10 mm MgCl_2_ and homogenized using a drill‐adapted pestle. Serial dilutions were plated on KB agar containing 25 μg/mL kanamycin and 25 μg/mL nystatin, and colonies were counted after 2 days of incubation at 28 °C. Equal inoculations with different bacterial strains were confirmed by serial dilution and colony counting of the initial infection suspensions.

### Quantification of cdG concentrations

The extraction and quantification of cdG were performed using high‐performance liquid chromatography‐coupled tandem mass spectrometry (HPLC‐MS/MS) analysis (Spangler *et al*., 2010). For cdG extraction, bacteria were grown on plates, as described for the production of bacterial cell extracts. Cells were scraped off and resuspended in 300 μL of ice‐cold extraction solvent, a mixture of acetonitrile–methanol–water (40 : 40 : 20, v/v/v), and incubated for 10 min at 4 °C to extract nucleotides. The cell suspension was heated to 95 °C for 10 min, cooled and centrifuged at 13 000 ***g*** for 5 min. The supernatant was stored and extraction of the resulting pellet was repeated twice with 200 μL of extraction solvent at 4 °C, omitting the heating step. The combined supernatants were evaporated until dryness at 40 °C in a miVac vacuum concentrator (Genevac, Ipswich, UK) and the dried residue was resuspended in 200 μL of water. The protein content was determined by dissolving the cell pellet in 200 μL of 0.1 m NaOH, and then heating for 15 min at 95 °C, before centrifugation and the measurement of protein concentration in the supernatant using a NanoDrop (Thermo Fisher Scientific, Waltham, MA, USA) at *λ* = 280 nm. Final cdG concentrations were expressed as pmoles per milligram of bacterial protein. Extractions were performed with two independent bacterial cultures as biological duplicates.

Nucleotide extracts were analysed by LC‐MS using an Acquity UPLC System attached to a TQS tandem mass spectrometer (Waters, Milford, MA, USA). Separation was performed on a Kinetix XB‐C18, 50‐mm × 2.1‐mm, 2.6‐μm column (Phenomenex, Torrance, CA, USA) using the following gradient of acetonitrile (solvent B) versus 0.1% formic acid in water (solvent A), run at 600 μL/min and 30 °C: 0 min, 1% B; 1 min, 1% B; 2.5 min, 25% B; 4 min, 70% B; 4.05 min, 1% B; 5.8 min, 1% B. The retention time for synthetic cdG was 1.21 min. Detection of cdG was by positive electrospray selected reaction monitoring (SRM) of the transition *m*/*z* 691/152 at a collision energy of 38 V and cone voltage of 50 V. The spray chamber conditions were as follows: capillary voltage, 1.8 kV; desolvation temperature, 600 °C; desolvation gas flow, 1000 L/h; cone gas flow, 150 L/h; nebulizer pressure, 7.0 bar. Quantification was by external standard calibration using standards from 10 to 5000 ng/mg.

### Measurement of ROS burst

The generation of ROS was measured as described previously (Schwessinger *et al*., 2011). Eight leaf discs (diameter, 4 mm) per *A. thaliana* genotype or *N. benthamiana* plant were collected into 96‐well plates and allowed to recover overnight in sterile water. The water was then replaced with a solution containing 17 mg/mL luminol (Sigma‐Aldrich, St. Louis, Missouri, USA), 200 μg/mL horseradish peroxidase (HRP) (Sigma‐Aldrich) and either bacterial extracts with 50 ng/mL protein concentration or 1 μm cdG (BioLog, Bremen, Germany) or 10 nm flg22 peptide (EZBiolab, Westfield, IN, USA) with the sequence described previously (Felix *et al*., 1999). Luminescence was recorded over a 60‐min time period using a charge‐coupled device camera (Photek Ltd., St Leonards on Sea, East Sussex, UK). Statistically significant differences between ROS outputs were determined by a two‐tailed Mann–Whitney test, and experiments were repeated at least twice independently in each case.

### Virus‐induced gene silencing

Virus‐induced gene silencing in *N. benthamiana* was performed using the *Tobacco rattle virus* (TRV) system, as described previously (Peart *et al*., 2002). The TRV‐RNA1 construct was contained in a pBINTRA6 vector and the TRV‐RNA2 constructs *TRV*:*GFP* and *TRV*:*SU* were located in a pTV00 vector (Peart *et al*., 2002). The *NbFls2* silencing construct was amplified from *N. benthamiana* cDNA using the primers 5′‐CGACGACAAGACCCTTACCTTTTTCATACCTTTG and 5′‐GAGGAGAAGAGCCCTGGTGGAATATTTCC, and subsequently cloned into the pYY13 vector (Dong *et al*., 2007). *Agrobacterium tumefaciens* GV3101 pMp90 containing the binary TRV‐RNA1 and TRV‐RNA2 constructs was resuspended in infiltration buffer consisting of 10 mm 2‐(*N*‐morpholino)ethanesulfonic acid (pH 5.5), 10 mm MgCl_2_ and 150 mm acetosyringone, and mixed in a 2 : 3 ratio (RNA1 : RNA2) with a final OD_600_ of 1.0. Two‐week‐old *N. benthamiana* plants were infiltrated with the *Agrobacterium* solution. Infection and systemic spread of the virus were monitored in *TRV:SU* plants, which were bleached as a result of reduced chlorophyll content on silencing. Silenced plants were used for experiments 3 weeks after infiltration. Successful silencing of *NbFls2* was examined in a ROS assay applying 10 nm of flg22 peptide.

### Immunoblot analysis

Bacterial extracts were separated on a 12% sodium dodecylsulfate polyacrylamide gel electrophoresis (SDS‐PAGE) gel and blotted on polyvinylidene difluoride (PVDF) membrane blocked with 5% milk in TBS with 0.1% [v/v] Tween 20. The flagellin protein was detected with a monoclonal α‐flagellin antibody specific to purified flagellin from *P. aeruginosa* (mabg‐flapa, Invivogen, Toulouse, France) used in a 1 : 1000 dilution and α‐mouse‐HRP (Sigma‐Aldrich) used in a 1 : 10 000 dilution as secondary antibody. Visualization was achieved with chemiluminescent ECL pico solution (Thermo Fisher Scientific) and imaged with a LAS 4000 ImageQuant system (GE Healthcare, Little Chalfont, Buckinghamshire, UK). Equal loading of protein was determined by Coomassie Brilliant Blue staining of the blotted membrane.

## Supporting information


**Fig. S1** *wspR19* overexpression leads to enhanced Congo Red dye binding (A) and reduced motility (B).

**Fig. S2** Illustration of the regulation of flagella synthesis by wspR19 expression.
