# Supplementary figures and images for: High levels of cyclic‐di‐GMP in plant‐associated P seudomonas correlate with evasion of plant immunity
Source: Mol Plant Pathol. 2015 Oct 8;17(4):521–31. doi: 10.1111/mpp.12297 (PMC4982027; doi:10.1111/mpp.12297)

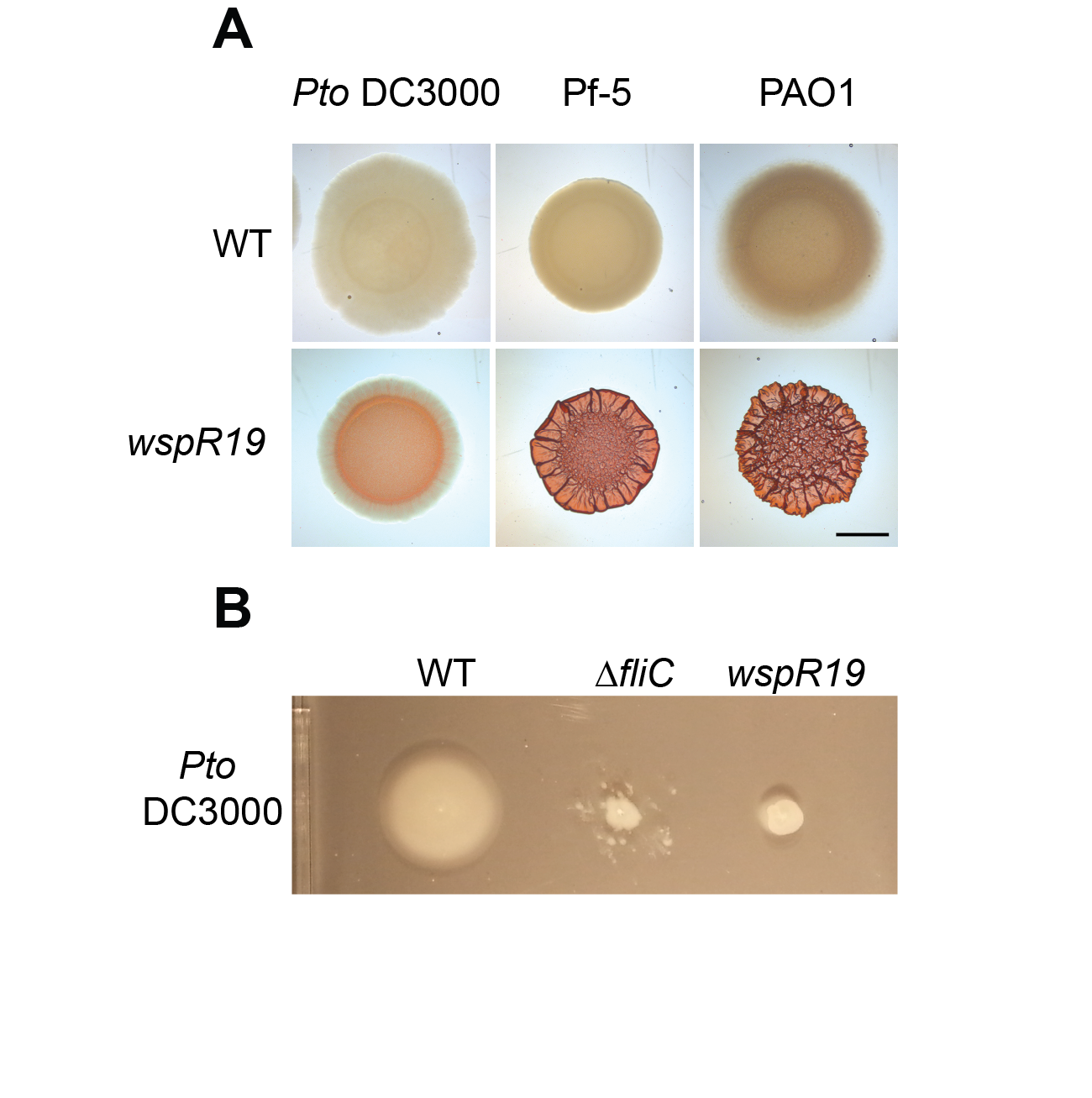

Supplement: Supplementary file 1 — Fig. S1 wspR19 overexpression leads to enhanced Congo Red dye binding (A) and reduced motility (B). [file MPP-17-521-s001.tif]

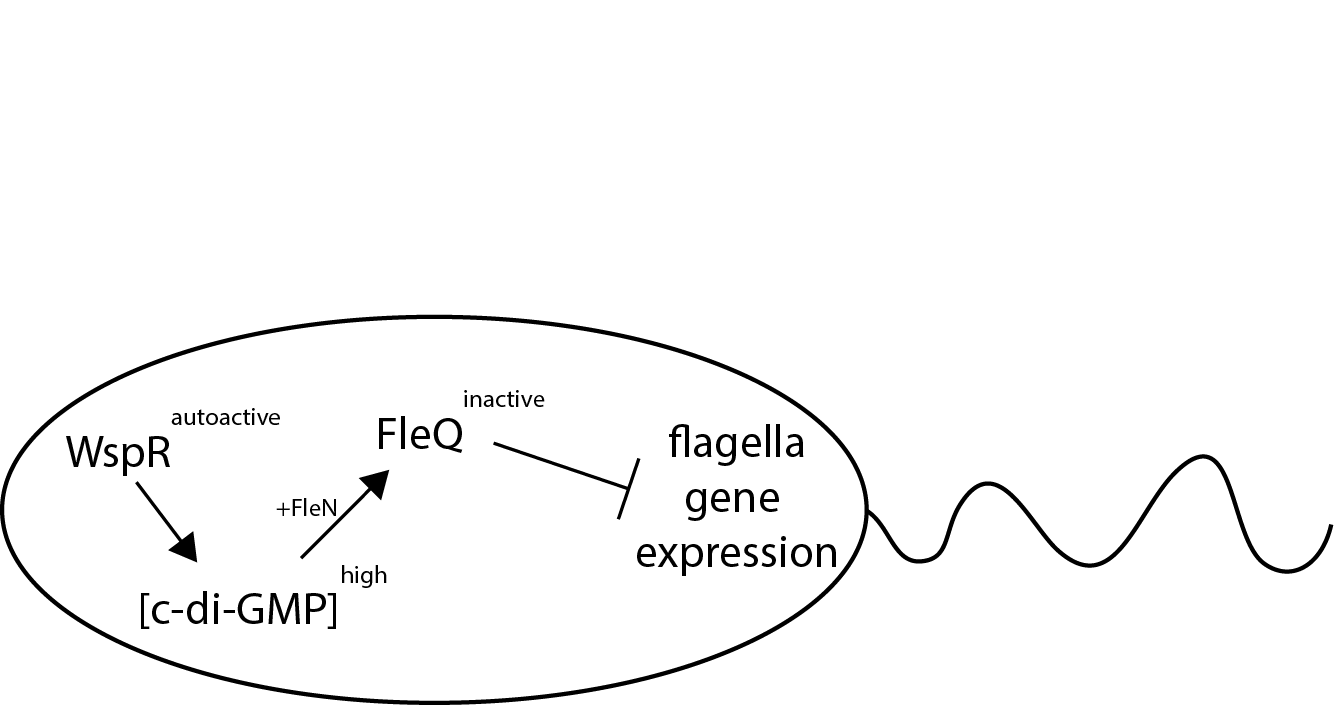

Supplement: Supplementary file 2 — Fig. S2 Illustration of the regulation of flagella synthesis by wspR19 expression. [file MPP-17-521-s002.tif]
